# *Fusobacterium nucleatum* in cancer: Interactions with microbiota, tumour colonisation and cancer progression

**DOI:** 10.1002/ctm2.70704

**Published:** 2026-05-29

**Authors:** Feng Zhao, Rui An, Shaobo Yu, Yilei Ma, Wangwang Liu, Caixia Sheng, Xinyou Xie, Jun Zhang

**Affiliations:** ^1^ Department of pathology Sir Run Run Shaw Hospital Zhejiang University School of Medicine Hangzhou Zhejiang China; ^2^ Key Laboratory of Precision Medicine in Diagnosis and Monitoring Research of Zhejiang Province Hangzhou Zhejiang China; ^3^ Department of Clinical Laboratory Sir Run Run Shaw Hospital Zhejiang University School of Medicine Hangzhou Zhejiang China

**Keywords:** chemoresistance, *Fusobacterium nucleatum*, immune evasion, targeted microbiome therapy, tumour microbiome, tumour microenvironment

## Abstract

**Background:**

Growing evidence from multi‐cancer cohort studies has positioned the oral pathobiont *Fusobacterium nucleatum* (*F. nucleatum*) as an emerging microbial contributor to cancer progression. Increased intratumoral abundance of *F. nucleatum* has been reported in colorectal, breast, esophageal, pancreatic, oral, and gastric cancers and is frequently associated with adverse clinicopathological features, treatment resistance, metastatic behavior, and poor prognosis. Advances in microbiome profiling, spatial analysis, and single‐cell technologies have begun to reveal how *F. nucleatum* colonizes tumors and interacts with host cells and tumor‐associated microbial communities.

**Main body:**

This review summarizes current evidence regarding the tumor‐associated activities of *F. nucleatum*, with emphasis on its routes of tumor entry, spatiotemporal colonization patterns, adhesion‐ and glycan‐dependent tropism, polymicrobial niche formation, and crosstalk with cancer cells and immune components. We discuss how *F. nucleatum* promotes oncogenic signaling, inflammatory amplification, genomic and epigenetic reprogramming, epithelial–mesenchymal transition, metastatic dissemination, immune evasion, and therapy adaptation. Particular attention is given to its context‐dependent effects on chemotherapy, radiotherapy, and immunotherapy responses, as well as emerging strategies aimed at detecting or selectively targeting intratumoral *F. nucleatum*.

**Conclusion:**

*F. nucleatum* represents both a biomarker‐associated organism and a potentially modifiable component of the tumor microenvironment. Defining its strain‐level heterogeneity, spatial ecology, and therapy‐specific functions will be essential for translating microbiome‐guided precision oncology from mechanistic insight into clinical application.

**Key points:**

- *F. nucleatum* colonises tumours through mucosal translocation, adjacent‐tissue migration and hematogenous dissemination.
- It promotes cancer progression via adhesion, inflammation, immune evasion, epigenetic remodelling and metastasis.
- Its effects on therapy response are tumour‐context dependent.
- Microbiome‐guided targeting may enable precision oncology.

## Graphical abstract

1. *F. nucleatum* colonises tumours through mucosal translocation, adjacent‐tissue migration and hematogenous dissemination.
2. It promotes cancer progression via adhesion, inflammation, immune evasion, epigenetic remodelling and metastasis.
3. Its effects on therapy response are tumour‐context dependent.
4. Microbiome‐guided targeting may enable precision oncology.

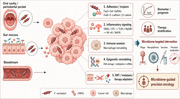

## 1 INTRODUCTION

**FIGURE 1:**
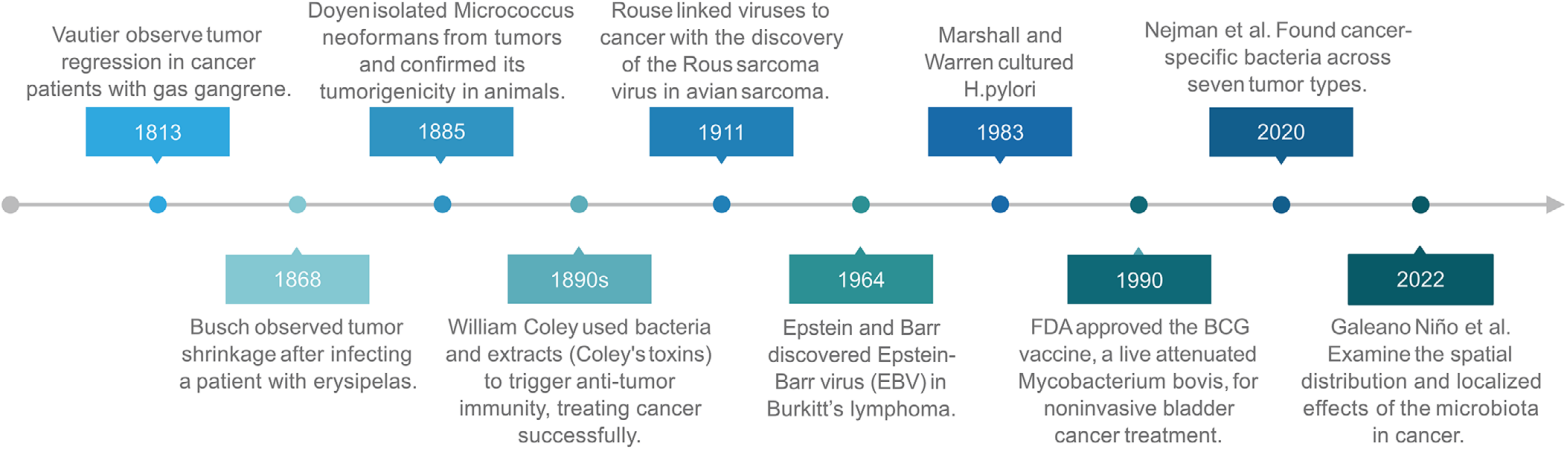
Milestones in intratumoural microbiome research. The key findings in intratumoural microbiome research and the major advancements in microbiome‐based anticancer therapies.

Over the past century, the relationship between microorganisms and cancer has evolved from a marginal observation into a major area of mechanistic and translational research (Figure 1). Early landmark discoveries, particularly those involving oncogenic viruses, first established the concept that microorganisms can actively contribute to carcinogenesis.^1^, ^2^, ^3^ These studies provided an important conceptual foundation for later investigations into bacteria–tumour interactions.

In contrast to the early emphasis on oncogenic viruses, bacteria entered cancer research through a more paradoxical route: they were initially explored as potential anticancer agents.^4^, ^5^, ^6^, ^7^ Historical observations of infection‐associated tumour regression inspired the development of bacterial immunotherapy, culminating in Coley's toxins and, later, the successful clinical use of Bacillus Calmette–Guérin for non‐muscle‐invasive bladder cancer.^8^, ^9^ These observations also highlighted that bacteria can shape antitumour immunity, reinforcing the idea that microbes are functionally relevant components of cancer biology rather than passive bystanders.

At the same time, accumulating evidence established that bacteria can also promote tumourigenesis. Landmark studies linking Helicobacter pylori to gastric cancer, observations from germ‐free models and sequencing‐based analyses of tumour‐associated microbiota collectively demonstrated that bacterial communities can influence proliferation, invasion, metastasis and immune escape within the tumour microenvironment (TME).^10^, ^11^, ^12^, ^13^, ^14^, ^15^, ^16^, ^17^, ^18^, ^19^, ^20^ More recently, spatial and single‐cell approaches have further shown that intratumoural bacteria, including *F. nucleatum*, preferentially localise to poorly vascularised and immunosuppressed niches, underscoring their active role in tumour ecology.^17^, ^21^, ^22^

Collectively, these advances have shifted the field from viewing microbes as incidental tumour inhabitants to recognising them as active regulators of oncogenesis, immune remodelling and therapeutic response. This conceptual transition has also reframed a central question in cancer microbiome research: how do specific microbial species, rather than the microbiota in general, drive distinct tumour phenotypes? In this context, *F. nucleatum* has emerged as one of the most compelling candidate organisms for mechanistic investigation.

Among the diverse microorganisms implicated in cancer, *F. nucleatum* has attracted particular attention because its biology is well characterised in oral disease and increasingly linked to tumour progression.^7^, ^17^, ^23^, ^24^ *F. nucleatum* is a well‐established oral pathobiont and a key pathogen in periodontitis and gingivitis.^25^, ^26^, ^27^ Within periodontal pockets and dental plaque, it functions as a bridging organism that promotes polymicrobial biofilm assembly, thereby aggravating tissue destruction and alveolar bone loss.^28^, ^29^, ^30^ Clinical studies have shown that its abundance correlates with disease severity, and interventions targeting *F. nucleatum* can mitigate periodontitis progression.^29^, ^31^, ^32^ These well‐established observations in oral disease provided the conceptual and biological basis for investigating its systemic dissemination and cancer‐associated roles. Subsequent studies detected *F. nucleatum* enrichment in multiple tumour types, including colorectal, pancreatic, oesophageal and breast cancers, where its presence is frequently associated with unfavourable clinical outcomes.^33^, ^34^, ^35^, ^36^, ^37^, ^38^, ^39^, ^40^, ^41^, ^42^ Mechanistically, *F. nucleatum* has been implicated in tumour colonisation, inflammatory signalling, immune evasion, metastasis and treatment resistance.^40^, ^43^, ^44^, ^45^, ^46^, ^47^, ^48^, ^49^ Emerging genomic evidence further indicates that strain/subspecies heterogeneity may shape these phenotypes, as illustrated by the distinct distribution of the Fna C1 and Fna C2 clades.^50^, ^51^, ^52^, ^53^ Notably, Fna C2 appears to display stronger colorectal cancer (CRC)‐associated virulence than Fna C1, promoting tumourigenesis in preclinical models through oxidative stress induction and metabolic reprogramming of the intestinal niche.^54^ Comparative genomic analyses further suggest that Fna C2 harbours genetic features that may facilitate niche adaptation and immune evasion.^54^ Given the growing clinical relevance of *F. nucleatum* in oncology,^55^ this review focuses on mechanistic basis of its tumour‐associated activities rather than attempting to comprehensively catalogue all microbiome–cancer associations. We discuss its routes of tumour colonisation, its oncogenic and immunomodulatory programs and its contribution to metastasis, treatment resistance and therapeutic response. By integrating these lines of evidence, we aim to clarify the translational significance of *F. nucleatum* as both a biomarker‐associated organism and a potential therapeutic target in cancer.

## 2 TUMOUR‐ASSOCIATED MICROBIOTA AND *F. NUCLEATUM* OVERVIEW

**FIGURE 2:**
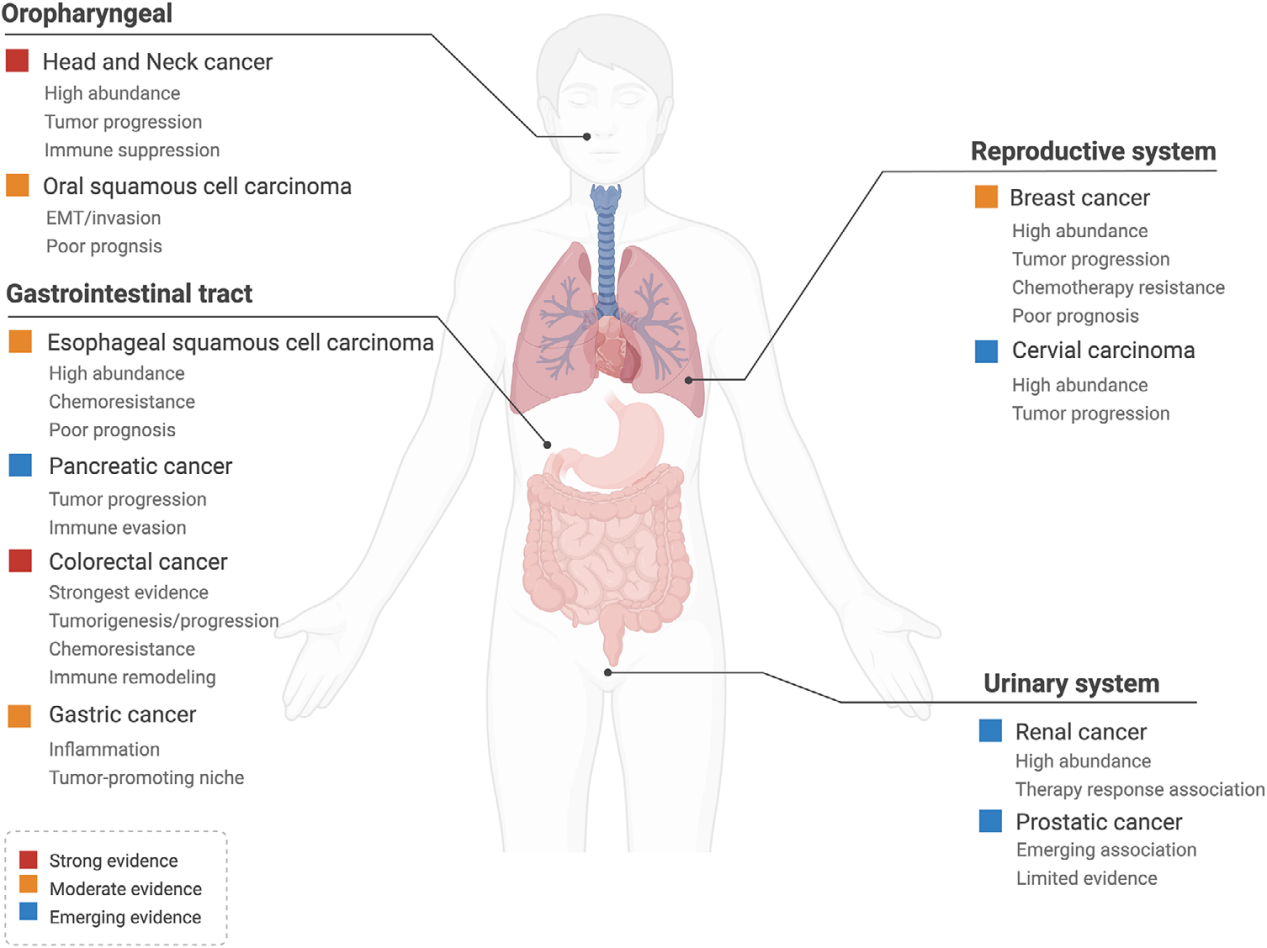
Cross‐cancer overview of *F. nucleatum* enrichment and functional associations. The figure summarises representative cancer types in which *F. nucleatum* has been reported to accumulate, together with major biological and clinical associations, including tumour progression, immune remodelling, metastasis, chemoresistance and therapy response. Evidence levels were assigned based on the amount and depth of available data: strong evidence indicates repeated human cohort data with mechanistic validation; moderate evidence indicates human association studies with limited mechanistic support; emerging evidence indicates preliminary, small‐cohort or indirect evidence. Representative references are indicated for each cancer type.

The human body harbours a vast and diverse microbial ecosystem, with the gastrointestinal (GI) tract serving as one of its principal reservoirs.^56^ Advances in next‐generation sequencing and other low‐biomass microbiome profiling approaches have substantially reshaped our understanding of host‐associated microbial communities. These technologies have revealed microbial signals in organs previously considered sterile, thereby challenging traditional assumptions about organ‐specific microbiology and raising new questions regarding the biological significance of tumour‐resident microbes.^17^ Among tumour‐associated microbes, *F. nucleatum* has been repeatedly detected at increased abundance in multiple malignancies, including colorectal, oral, breast, oesophageal and other GI cancers, prompting intensive investigation into its role in carcinogenesis and tumour progression.^22^, ^39^, ^40^, ^57^, ^58^ Recent studies have further expanded the spectrum of implicated tumour types. In oral cancer, *F. nucleatum*‐derived outer membrane vesicles (OMVs) were shown to promote metastasis by activating autophagic flux, whereas in gastric cancer, intratumoural *F. nucleatum* was reported to recruit tumour‐associated neutrophils and facilitate immune evasion through NF‐κB‐related signalling.^59^, ^60^ Mechanistically, *F. nucleatum* appears to influence cancer progression through several interconnected processes, particularly by amplifying inflammatory signalling that creates a microenvironment permissive for tumour initiation and progression.^49^, ^61^, ^62^, ^63^, ^64^ In parallel, *F. nucleatum* can suppress antitumour immunity by impairing immune‐cell function, reshaping immune‐cell composition and enhancing immune‐evasive phenotypes in tumour cells. It may also remodel the physicochemical properties of the TME, including local oxygenation, nutrient availability and niche architecture, thereby supporting tumour growth, persistence and metastatic dissemination. In addition, accumulating studies suggest that *F. nucleatum* can influence host‐cell transcriptional, genetic and epigenetic programs, thereby modifying malignant phenotypes and potentially contributing to intertumoural heterogeneity.^45^, ^65^, ^66^ Rather than acting in isolation, *F. nucleatum* may cooperate with other members of the tumour microbiome to establish polymicrobial communities or biofilm‐like niches that reinforce inflammation, immune suppression and malignant progression. Emerging evidence also links *F. nucleatum* to resistance or altered responsiveness to chemotherapy, radiotherapy and immunotherapy, highlighting its potential relevance as both a biomarker‐associated organism and a therapeutic target (Figure 2).^44^, ^65^, ^67^, ^68^, ^69^, ^70^ Collectively, current evidence suggests that *F. nucleatum* exerts both shared and context‐dependent effects across tumour types: while inflammatory activation, immune evasion and therapeutic modulation emerge as recurring themes, the dominant cellular targets and downstream pathways appear to vary according to tissue ecology and microbial context.

## 3 TUMOUR COLONISATION ROUTES AND SPATIOTEMPORAL DYNAMICS

### 3.1 Sources/routes

**FIGURE 3:**
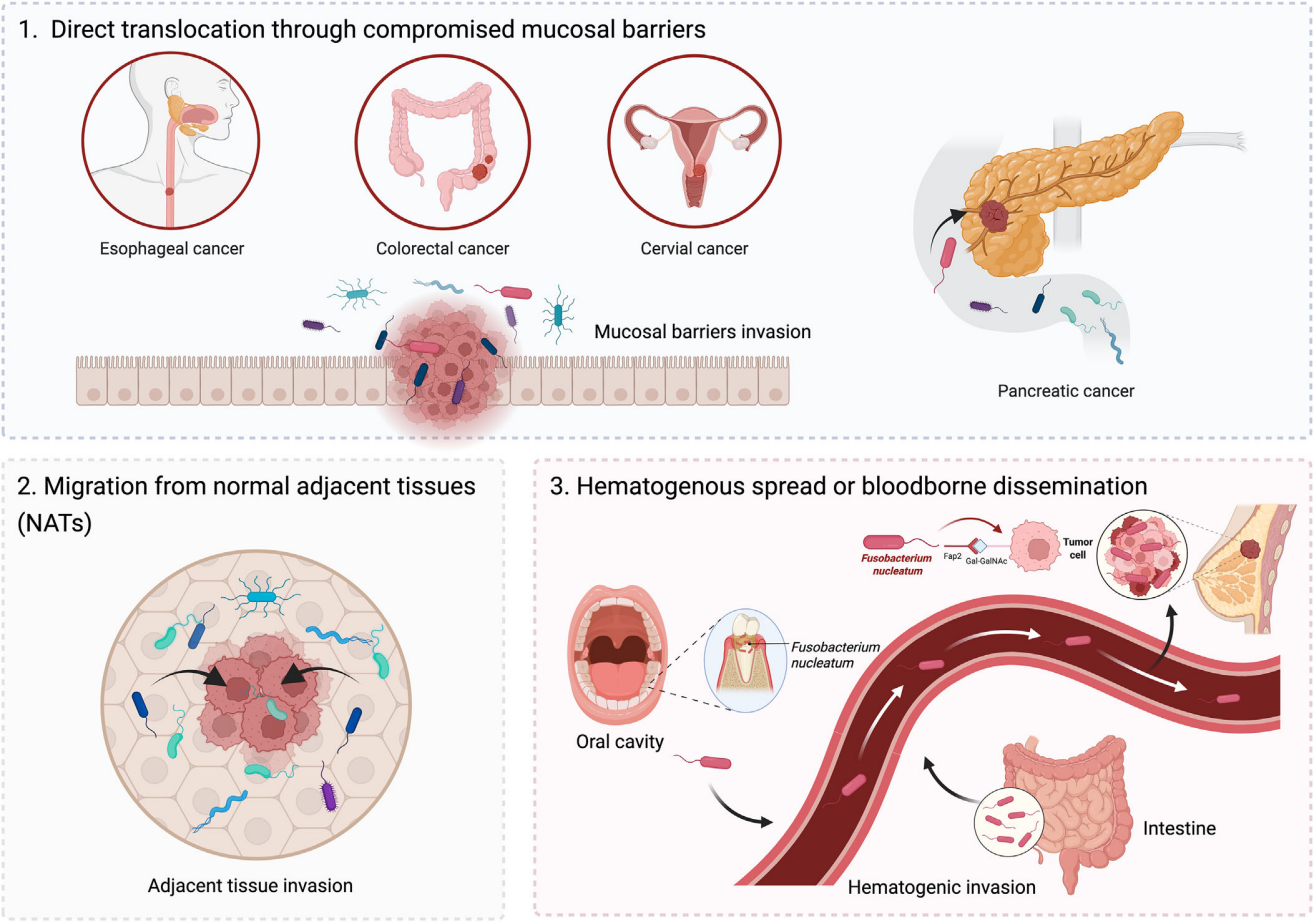
Proposed routes of microbial entry into tumours. Three non‐mutually exclusive pathways may account for the presence of intratumoural microbes: (1) direct translocation across compromised mucosal barriers; (2) migration from normal adjacent tissues (NATs), based on microbial similarity between tumours and nearby non‐malignant tissues; and (3) hematogenous dissemination, in which bacteria originating from sites such as the oral cavity or gut enter the circulation and seed susceptible tumours.

Despite growing recognition of the importance of intratumoural microbiota in oncology, their precise origins and routes of tumour entry remain incompletely defined. Walker et al. highlighted several tumour‐associated conditions that may facilitate bacterial colonisation, including disorganised vasculature, a nutrient‐rich yet hypoxic milieu and localised immune suppression.^71^ Based on the ecological features of the TME and current evidence on bacterial dissemination, three non‐mutually exclusive routes may account for microbial entry into tumours: (1) direct translocation across compromised mucosal barriers; (2) migration from normal adjacent tissues (NATs); and (3) hematogenous dissemination through the circulation (Figure 3).

Mucosal organs are major microbial reservoirs, and tumours arising in these sites – such as colorectal, pancreatic, cervical, oesophageal and pulmonary malignancies – may permit microbial entry through barrier disruption during tumourigenesis or other pathological processes.^72^, ^73^, ^74^ In CRC, multiple studies have isolated viable *F. nucleatum* from tumour biopsy specimens and, using patient‐derived xenograft models, provided evidence supporting an oral origin for these intratumoural bacteria.^34^, ^46^, ^75^, ^76^ However, a mucosal‐origin model alone may not fully explain bacterial signals detected in non‐mucosal tumours such as breast cancer. In these contexts, the detected taxa are not always typical of the most obvious source mucosa, suggesting that additional routes – particularly hematogenous seeding or migration from adjacent tissue reservoirs – may also contribute. Consistent with this possibility, a 2020 study proposed that some intratumoural bacteria may arise from NATs on the basis of microbial similarity between tumours and adjacent normal tissues.^17^ Although the authenticity and relative contribution of NAT‐derived microbes remain to be established, this model provides a plausible explanation for bacterial signals in tissues not readily accounted for by direct mucosal translocation.^77^, ^78^

Hematogenous dissemination provides a second major route by which microbes may access distant tumour sites. Once in the circulation, microbes may preferentially seed tumours because necrotic debris, vascular leakiness and local immune dysfunction create a permissive colonisation niche.^19^, ^79^ The oral cavity is a particularly plausible reservoir for this process, as oral bacteria can enter the bloodstream and subsequently colonise distant anatomical sites, including breast tissue.^40^ Dental procedures, routine oral hygiene and especially periodontal disease have all been implicated in facilitating the transient entry of oral microbes into the systemic circulation.^80^, ^81^, ^82^ During these episodes of bacteraemia, circulating bacteria may gain access to secondary niches in susceptible tissues.^83^, ^84^, ^85^, ^86^, ^87^ In immunocompetent individuals, such episodes of bacteraemia are usually transient and effectively cleared. However, in the setting of malignancy – particularly when vascular integrity is compromised or host immunity is suppressed – these circulating bacteria may encounter conditions favourable for persistent tumour colonisation. In this context, *F. nucleatum* possesses several traits that may enhance tumour seeding. Its adhesin FadA can bind E‐cadherin on endothelial cells, activate β‐catenin signalling, disrupt intercellular junctions and thereby facilitate trans‐endothelial passage into tumour tissue. In parallel, Fap2 promotes attachment to cancer cells expressing Gal–GalNAc, potentially reinforcing retention at the tumour site.^40^ Consistent with this model, Abed et al. compared intravenous and oral inoculation in orthotopic CRC mouse models and found that intravenous delivery resulted in more efficient tumour colonisation than oral administration.^76^ These findings support the view that hematogenous dissemination is not merely possible, but may represent an efficient route for tumour seeding by *F. nucleatum* under selected conditions.

Defining the relative contribution of these routes is important not only for understanding tumour microbial ecology, but also for improving prevention, detection and therapeutic targeting strategies. Comparative analyses across tumours, adjacent tissues and candidate source sites may help identify the dominant route of entry in different cancers. More broadly, elucidating how microbes access and persist within the TME remains a key prerequisite for translating tumour microbiome biology into clinical application.

### 3.2 Establishment and biofilm polymicrobial niche

**FIGURE 4:**
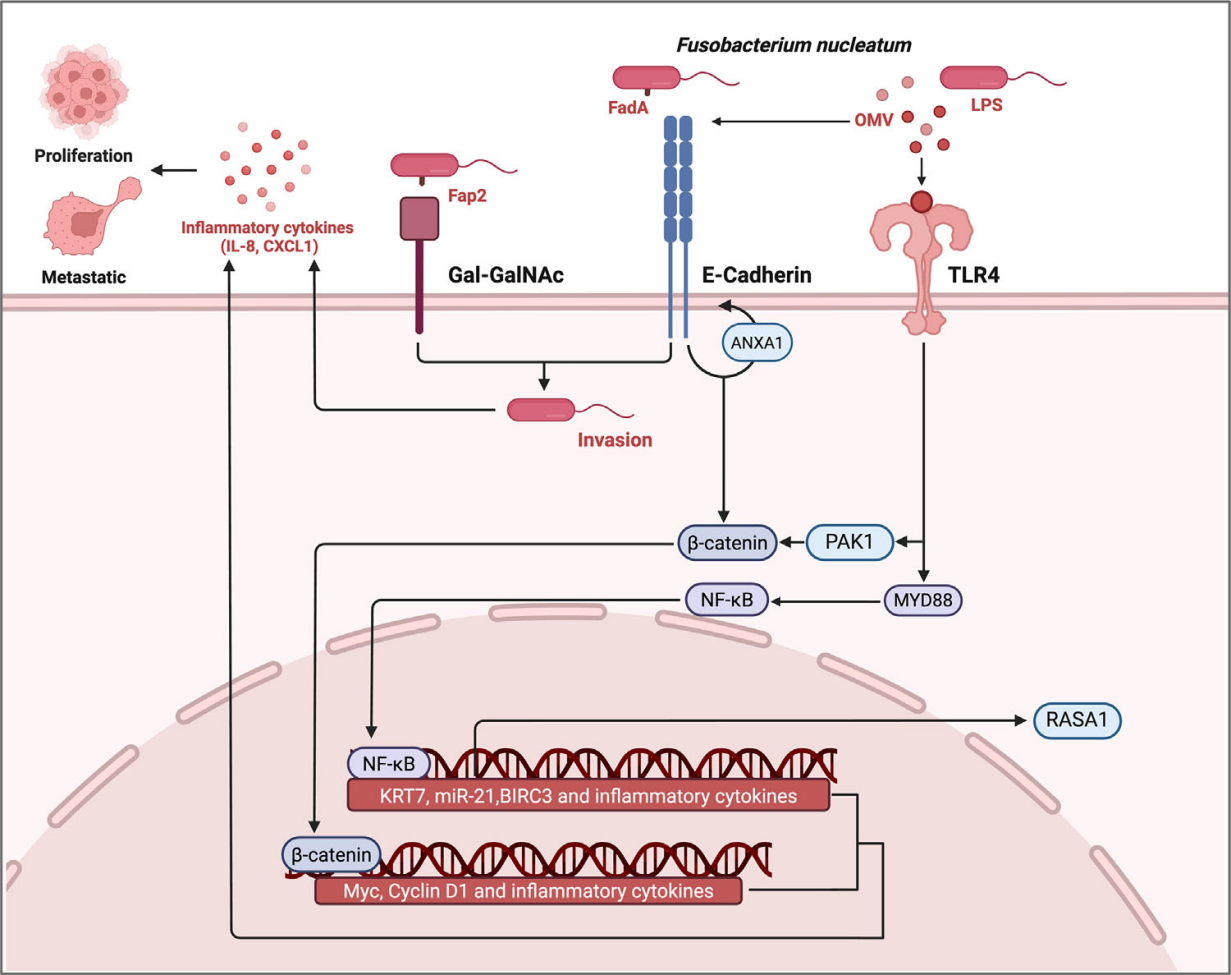
Oncogenic mechanisms of *F. nucleatum* in cancer. *F. nucleatum* promotes tumour progression through coordinated adhesion‐dependent and inflammatory signalling programs. Fap2‐mediated recognition of the tumour‐associated glycan Gal–GalNAc facilitates bacterial attachment to malignant cells, whereas FadA engagement of E‐cadherin disrupts cell–cell adhesion and promotes β‐catenin accumulation and nuclear translocation. Activated β‐catenin signalling induces oncogenic transcriptional programs, including MYC, Cyclin D1 and inflammatory mediators. In parallel, *F. nucleatum*‐derived outer membrane vesicles (OMVs) and lipopolysaccharide (LPS) can activate Toll‐like receptor (TLR)‐dependent pathways, leading to MYD88/NF‐κB activation, miR‐21 induction, repression of the Ras negative regulator RASA1 and amplification of inflammatory cytokine production. Arrows indicate activation or up‐regulation, whereas T‐bar lines indicate inhibition or down‐regulation.

Once *F. nucleatum* reaches the tumour site, successful colonisation depends on its ability to adhere, persist and integrate into the local tumour ecosystem. In this setting, *F. nucleatum* can interact directly with cancer cells and indirectly remodel the surrounding inflammatory and immune milieu, thereby creating conditions favourable for persistent colonisation and tumour progression. A central step in this process is microbial adhesion. Through virulence factors such as Fap2, which recognises the tumour‐associated glycan Gal–GalNAc that is enriched in colorectal and breast cancers, *F. nucleatum* can preferentially attach to malignant cells and initiate downstream oncogenic and pro‐metastatic signalling.^40^, ^44^, ^47^, ^67^, ^88^, ^89^, ^90^, ^91^, ^92^ Beyond surface attachment, accumulating evidence suggests that tumour‐associated bacteria may also persist within cancer cells or other protected intratumoural niches, where they are less accessible to host clearance and can sustain long‐range host–microbe signalling. This intracellular or sheltered localisation may be particularly relevant for explaining prolonged biological effects despite low absolute bacterial biomass. Colonisation is further stabilised by the formation of polymicrobial communities. Dual‐RNA sequencing and fluorescence in situ hybridisation studies have shown that *F. nucleatum* forms mixed‐species biofilms within colorectal tumours, particularly in necrotic regions and ulcerated mucosal surfaces.^22^, ^93^, ^94^, ^95^ These biofilm‐like structures are frequently co‐localised with other tumour‐associated bacteria, including *Bacteroides fragilis*, and are associated with increased expression of inflammatory mediators such as IL‐6, CXCL8 and MMP1, thereby promoting neutrophil recruitment and pro‐tumourigenic immune remodelling. Adhesion‐associated factors including FadA, Fap2 and RadD likely contribute to this process by facilitating bacterial aggregation, tissue retention and sustained host interaction.^22^, ^93^, ^94^, ^95^ Together, these findings indicate that tumour colonisation by *F. nucleatum* is not a passive event, but an active ecological process involving selective adhesion, niche adaptation, microbial cooperation and persistent inflammatory reinforcement (Figure 4).

## 4 ONCOGENIC MECHANISMS AND SIGNALLING PROGRAMS DRIVEN BY *F. NUCLEATUM*

Collectively, current evidence indicates that *F. nucleatum* promotes tumour progression through several interconnected oncogenic programs rather than a single dominant pathway. These programs include selective adhesion and glycan recognition, genomic and epigenetic reprogramming, activation of inflammatory and survival signalling, enhancement of EMT and metastatic dissemination and remodelling of the immune and vascular microenvironment. Although many of these mechanisms have been best characterised in CRC, emerging data suggest that similar principles may operate in breast, oral, oesophageal, pancreatic and other cancers in a context‐dependent manner.

### 4.1 Adhesion, glycan tropism and genomic/epigenetic reprogramming

A prominent oncogenic consequence of *F. nucleatum* infection is host genomic and epigenetic reprogramming. In CRC, *F. nucleatum* has been linked to increased DNA methyltransferase activity and hypermethylation of tumour suppressor gene promoters, thereby contributing to CpG island methylator phenotype (CIMP), microsatellite instability‐high (MSI‐H) status and other molecular alterations associated with aggressive disease.^96^, ^97^, ^98^ Consistent with this, multiple studies have reported associations between high intratumoural *F. nucleatum* levels and CIMP, MSI‐H, BRAF mutations, hMLH1 methylation and additional gene alterations in CRC tissues.^41^, ^99^, ^100^, ^101^

**FIGURE 5:**
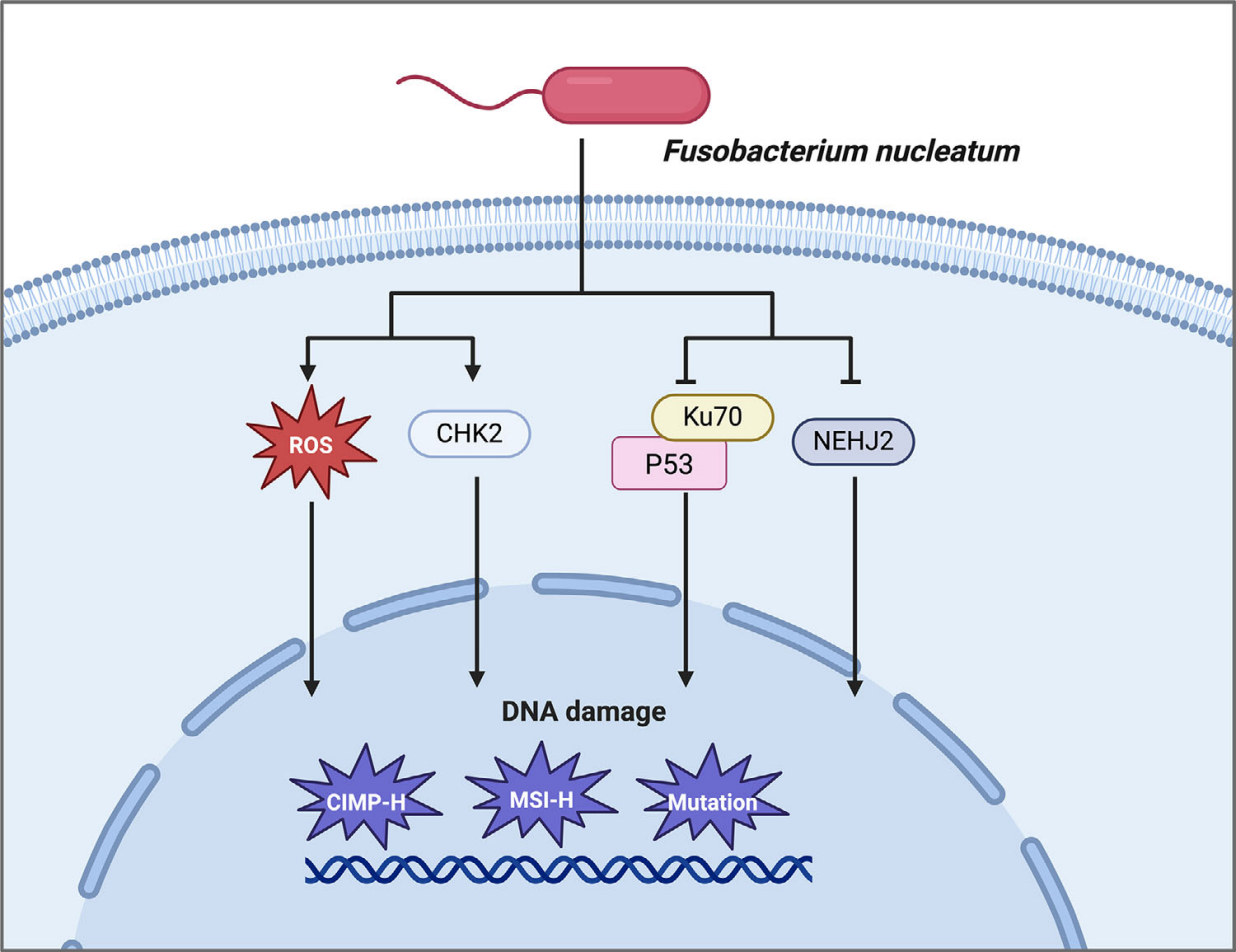
Potential mechanisms linking *F. nucleatum* to DNA damage and genomic instability in cancer. *F. nucleatum* has been associated with DNA damage, defective DNA repair and genomic instability in cancer, particularly in colorectal cancer. Mechanistically, *F. nucleatum* may increase reactive oxygen species (ROS), thereby promoting oxidative stress and DNA lesions. It has also been reported to affect DNA damage‐response and repair‐associated pathways, including Chk2 checkpoint signalling, the Ku70/p53 axis and non‐homologous end joining (NHEJ). These alterations may contribute to DNA damage accumulation and have been linked to molecular features such as CpG island methylator phenotype‐high (CIMP‐H), microsatellite instability‐high (MSI‐H) and increased mutation burden. Because some associations remain incompletely resolved, this model summarises plausible and reported mechanisms rather than implying that all downstream genomic alterations are directly caused by *F. nucleatum*. Arrows indicate activation or up‐regulation, whereas T‐bar lines indicate inhibition or down‐regulation.

Beyond promoter hypermethylation, *F. nucleatum* may also contribute to mutational burden and impaired DNA‐damage control. CRCs with high *F. nucleatum* abundance have been reported to harbour more transition mutations and higher frequencies of mutations in genes such as APC and ATM, although the causal pathways linking bacterial colonisation to these alterations remain incompletely resolved.^102^ One plausible mechanism is that persistent inflammatory signalling increases reactive oxygen species (ROS), thereby enhancing genomic stress; however, direct evidence linking ROS‐mediated damage to broad hypermethylation patterns remains limited.^98^ Additional studies suggest that *F. nucleatum* may interfere with checkpoint and repair pathways, including Chk2 signalling, the Ku70/p53 axis and the DNA glycosylase NEIL2, thereby promoting double‐strand breaks, defective repair and inflammation‐associated tumour progression.^103^, ^104^, ^105^ Collectively, these findings support a model in which *F. nucleatum* facilitates tumourigenesis not only through inflammatory activation, but also through sustained genomic instability and impaired damage repair (Figure 5).

Importantly, the epigenetic consequences of *F. nucleatum* infection extend beyond DNA methylation alone. Recent evidence indicates that *F. nucleatum* can influence histone‐modification states, including H3K27 acetylation, thereby reinforcing oncogenic transcriptional programs in hypoxic CRC microenvironments.^106^ In parallel, hypoxia may facilitate bacterial persistence while reshaping host non‐coding RNA networks, such as miR‐4802 and LINC01089, to support survival signalling and chemoresistance.^96^, ^98^, ^107^, ^108^, ^109^ These observations suggest a bidirectional interaction in which the hypoxic TME promotes bacterial adaptation, while *F. nucleatum* in turn strengthens epigenetic and transcriptional states favourable to malignant progression.

### 4.2 TLR signalling, inflammatory amplification and chemoresistance

#### 4.2.1 TLR4/MyD88 as a central signalling hub

**FIGURE 6:**
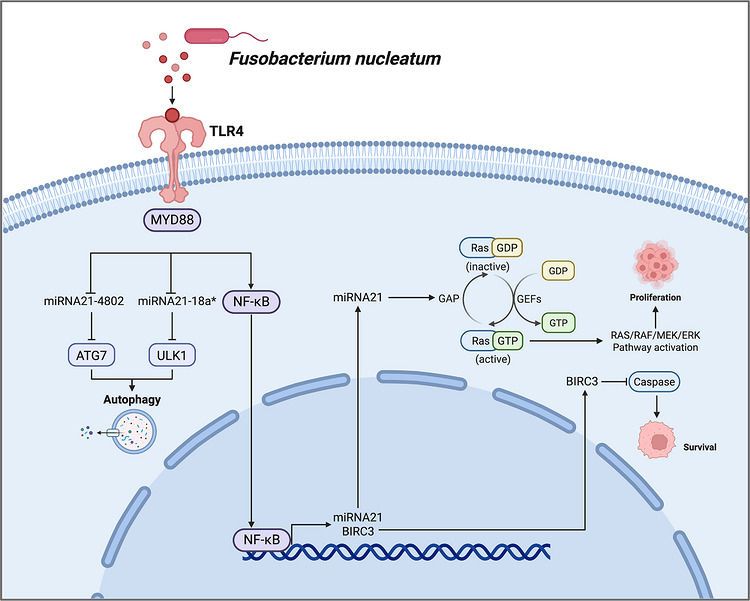
TLR4/MyD88‐centred signalling activated by *F. nucleatum* in cancer. *F. nucleatum* activates TLR4/MYD88‐centred signalling in tumour cells, particularly in colorectal cancer, thereby coordinating inflammatory activation, survival signalling, autophagy and therapy resistance. Engagement of TLR4 triggers MYD88‐dependent downstream pathways, including NF‐κB activation and transcriptional induction of inflammatory and survival‐related genes. This signalling has been linked to down‐regulation of autophagy‐suppressive miRNAs, including miR‐4802 and miR‐18a*, leading to increased ATG7 and ULK1 expression and enhanced autophagy. In parallel, *F. nucleatum*‐induced miR‐21 and related survival pathways may promote BIRC3 expression, inhibit caspase‐dependent apoptosis and activate RAS/RAF/MEK/ERK signalling, thereby supporting tumour‐cell proliferation, survival and reduced chemosensitivity. Arrows indicate activation or up‐regulation, whereas T‐bar lines indicate inhibition or down‐regulation.

The TLR4/MyD88 axis represents one of the best‐characterised signalling hubs through which *F. nucleatum* promotes tumour progression and treatment resistance, particularly in CRC.^47^, ^49^, ^110^ As a pattern‐recognition receptor, TLR4 senses bacterial components and initiates downstream signalling cascades that converge on NF‐κB, PI3K, MAPKs and interferon‐regulatory pathways.^111^ In multiple tumour types, including CRC and breast cancer, elevated TLR4 expression has been linked to invasion, migration, metastasis and poor clinical behaviour.^112^, ^113^, ^114^ Thus, *F. nucleatum*‐dependent engagement of TLR4 is best viewed not as an isolated event, but as a central upstream trigger that coordinates inflammatory activation, survival signalling, apoptosis resistance and therapy adaptation (Figure 6)

#### 4.2.2 NF‐κB‐driven inflammatory amplification

Chronic inflammatory signalling provides an important mechanistic bridge between bacterial persistence and malignant progression. *F. nucleatum* can activate NF‐κB‐centred inflammatory programs, often downstream of TLR engagement, resulting in increased production of cytokines and chemokines that support tumour growth, immune remodelling and metastatic behaviour.^44^, ^45^, ^47^, ^115^, ^116^, ^117^, ^118^, ^119^, ^120^, ^121^, ^122^, ^123^ Sustained NF‐κB activation is particularly relevant because it links microbial sensing to transcriptional programs governing proliferation, survival, differentiation and inflammatory reinforcement.

#### 4.2.3 Key cytokine effectors: IL‐6, IL‐8 and IL‐1β

Among the downstream mediators induced by *F. nucleatum*, IL‐6 appears to be particularly important. In CRC, *F. nucleatum* is associated with increased IL‐6 expression together with other inflammatory cytokines, whereas in breast cancer IL‐6 is already recognised as a poor‐prognosis factor linked to EMT and stromal remodelling.^45^, ^90^, ^124^, ^125^, ^126^, ^127^, ^128^, ^129^, ^130^ In addition, *F. nucleatum* can stimulate IL‐6 secretion from immune cells such as B lymphocytes and macrophages, further amplifying paracrine signalling within the TME.^119^, ^131^ This is biologically relevant because IL‐6/STAT3 activation in *F. nucleatum*‐infected tumour cells has been linked to EMT and cancer stem cell‐like phenotypes.^132^

**FIGURE 7:**
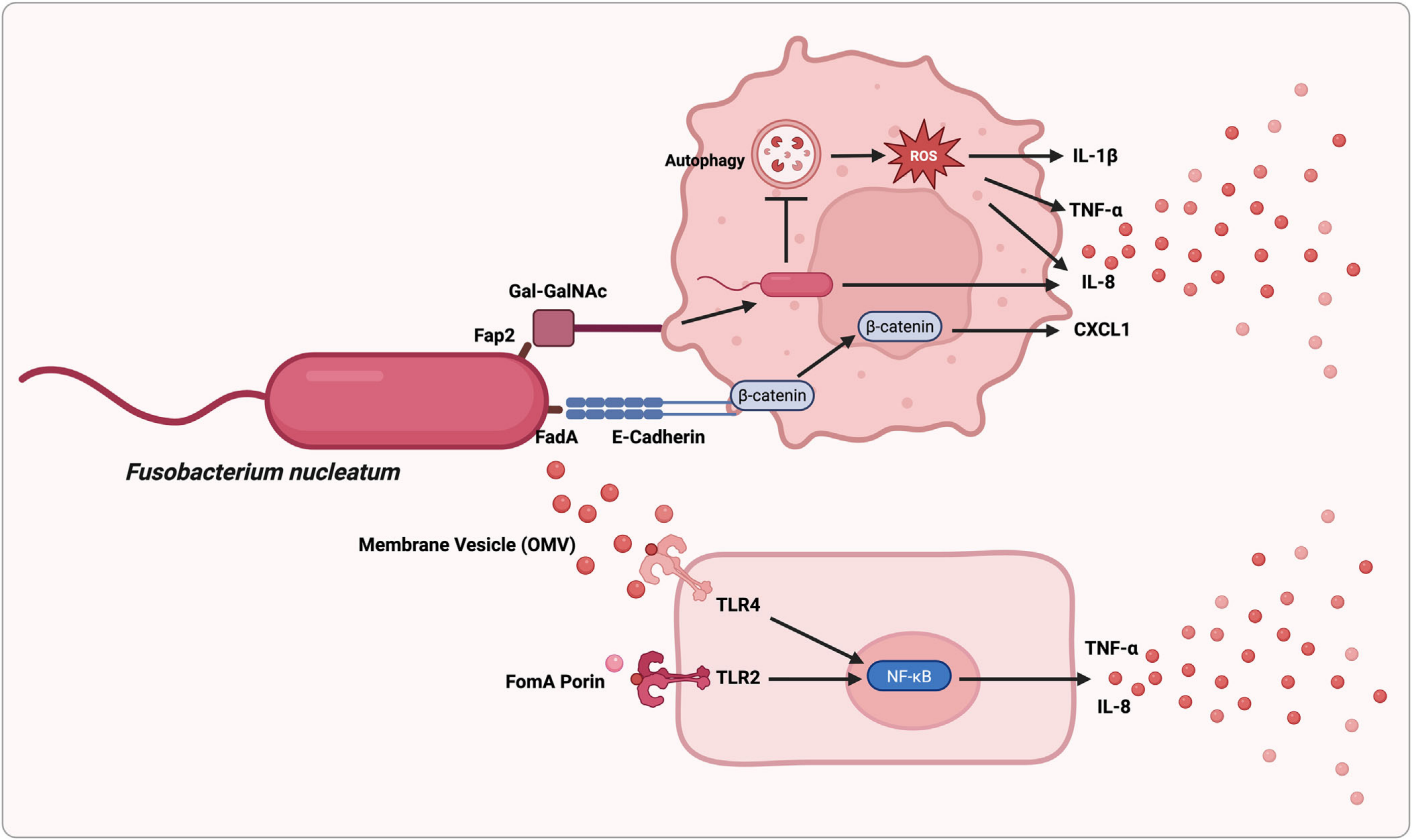
Representative inflammatory pathways activated by *F. nucleatum* in CRC. In colorectal cancer (CRC), *F. nucleatum* activates multiple inflammatory and invasion‐associated pathways through both direct bacterial–tumour cell contact and vesicle‐mediated signalling. FadA‐mediated interaction with host adhesion/signalling complexes promotes β‐catenin‐related signalling, reactive oxygen species (ROS) accumulation and production of pro‐inflammatory mediators, including IL‐1β, IL‐8, TNF‐α and CXCL1. In parallel, *F. nucleatum*‐derived outer membrane vesicles (OMVs) and the porin protein FomA can stimulate TLR2/TLR4 signalling, resulting in NF‐κB activation and transcriptional amplification of inflammatory genes. Arrows indicate activation or up‐regulation, whereas T‐bar lines indicate inhibition or down‐regulation.

IL‐8 represents another recurrent effector downstream of *F. nucleatum* signalling and has been associated with lymph node positivity and advanced‐stage disease in breast cancer.^133^, ^134^ Mechanistically, *F. nucleatum* can induce IL‐8 through TLR2/TLR4‐dependent signalling, NF‐κB activation, OMV‐ and FomA‐mediated stimulation, ROS accumulation, β‐catenin activation and FadA‐dependent invasion pathways.^43^, ^44^, ^90^, ^131^, ^135^, ^136^, ^137^ This convergence of distinct upstream inputs on a shared pro‐inflammatory output underscores how *F. nucleatum* amplifies tumour‐promoting cytokine circuits (Figure 7).

IL‐1β provides a further example of how *F. nucleatum* couples microbial sensing to inflammatory oncogenesis. Current evidence suggests that IL‐1β maturation is promoted through coordinated activation of inflammasome signalling and TLR4‐dependent transcriptional priming. FadA‐mediated disruption of E‐cadherin/β‐catenin complexes may increase mitochondrial ROS and favour NLRP3 inflammasome assembly, whereas OMV‐delivered bacterial components activate TLR4/MyD88/NF‐κB signalling and enhance IL1B transcription.^135^, ^138^, ^139^ These signals converge on caspase‐1‐dependent processing of pro‐IL‐1β into its mature form. In breast cancer models, *F. nucleatum*‐infected tumour‐associated macrophages display increased IL‐1β secretion, which can promote EMT in malignant cells through IL‐1R/MyD88/ERK signalling.^107^, ^135^, ^138^, ^140^ Taken together, IL‐1β appears to function as a mechanistic link between bacterial persistence, macrophage activation and malignant progression.

### 4.3 Immune evasion and immunosuppressive remodelling

The studies summarised in Table 1 provide strong evidence that *F. nucleatum* can reshape both the composition and function of immune‐cell populations within the TME, thereby fostering an immunosuppressive niche that favours tumour immune evasion. Rather than acting through a single pathway, *F. nucleatum* appears to suppress antitumour immunity through multiple coordinated mechanisms, including recruitment of immunosuppressive myeloid populations, enhancement of immune checkpoint‐associated molecules such as PD‐L1 and CD47 and induction of matrix‐remodelling factors with immunoregulatory activity.^44^, ^141^, ^142^, ^143^, ^144^, ^145^, ^146^

**TABLE 1:** Impact and mechanisms of *F. nucleatum* on immune cell function.

Cell type	Cancer type	Model	Effect	Mechanism	References
Peripheral blood lymphocytes	Not cancer‐specific	Lymphocyte cells (human)	Inhibition	Modifies DNA, RNA and protein synthesis	^147^
	Not cancer‐specific	Mononuclear cells (human)	Reduction	Induces apoptotic cell death	^148^
CD3+ T lymphocytes	Not cancer‐specific	T lymphocyte cell line (human)	Replication inhibition	Prevents entry into the G0/G1 phase of the cell cycle	^149^
	Not cancer‐specific	T lymphocyte cell line (human)	Reduction	Induces cell death via Fap2 and RadD proteins	^150^
	CRC	CRC tumour tissue (human)	Reduction	Mechanism not identified	^151^
CD4+ T lymphocytes	CRC	CRC model (mouse)	No change observed	Mechanism not identified	^45^
	CRC	CRC tumour tissue (human)	Reduction	Decreases T lymphocyte developmental protein TOX expression	^152^
	CRC‐related tumour‐immune model	CRC lymphocyte cell line (human)	Inhibition	Interaction between human TIGIT and Fap2	^153^
	Not cancer‐specific	CD4+ cells (human)	Inhibition	Activates CEACAM1	^154^
	Not cancer‐specific	CD4+ cells (human)	Inhibition	Binds to and activates CEACAM1 via CbpF	^155^
	BC	BC model (mouse)	Reduction	Mechanism not identified	^40^
	OSCC	OSCC tumour tissue (human)	Reduction	Mechanism not identified	^156^
CD8+ T lymphocytes	CRC	CRC model (mouse)	No change observed	Mechanism not identified	^45^
	CRC‐related tumour‐immune model	CRC lymphocyte cell line (human)	Inhibition	Interaction between human TIGIT and Fap2	^153^
	Not cancer‐specific	CD8+ cells (human)	Inhibition	Activates CEACAM1	^154^
	BC	BC model (mouse)	Reduction	Mechanism not identified	^40^
	ESCC	ESCC tumour tissue and cell line (human)	Inhibition	Enhances expression of CD8+ cell surface inhibitory receptor KIR2DL1	^157^
B lymphocytes	OSCC	OSCC tumour tissue (human)	Reduction	Mechanism not identified	^156^
T‐regulatory lymphocytes (TREGS)	ESCC	ESCC tumour tissue (human)	Increase	Mechanism not identified	^158^
	Non‐cancer intestinal model	Human intestine tissue and mouse models	Increase	Stimulates Toll‐like receptors 2 and 4	^159^
TH17 T lymphocytes	CRC	CRC model (mouse)	Increase	Through a FFAR2 (SCFA receptor) dependent manner	^28^
Natural killer cells	Non‐tumour mouse model	Mouse model	Reduced colonic NK cell activity and frequency	Mechanism not identified	^160^
	CRC‐related tumour‐immune model	CRC natural killer cell line	Inhibition	Interaction between human TIGIT and Fap2	^153^
	Not cancer‐specific	NK cells (human)	Inhibition	Activates CEACAM1	^154^
Macrophages	OSCC	OSCC tumour tissue (human)	Reduction in M2 macrophages	Mechanism not identified	^156^
	CRC	CRC tumour tissue and cultured macrophages (human and mouse)	Promotes M2 polarisation	Via the TLR4/IL‐6/p‐STAT3/c‐MYC pathway	^156^
	CRC	CRC tumour tissue (human)	Increase	Mechanism not identified	^161^
	CRC	CRC tumour tissue and patient faeces	Increased macrophage infiltration and M2 polarisation	Via CCL20 activation	^162^
	CRC	CRC tumour tissue (human)	Promotes M2 polarisation	Activates the TLR4/NF‐κB/S100A9 cascade	^163^
	Not cancer‐specific	Macrophage cell line	Promotes M1 polarisation	AI‐2 activates the TNFSF9/IL‐1β pathway	^164^

#### 4.3.1 Inhibition of NK and T Cells

Immune evasion is a crucial feature of cancer progression, and *F. nucleatum* contributes to this process in part by impairing the cytotoxic functions of NK cells and T cells. NK cells are essential components of innate immunity and play a major role in eliminating infected and malignant cells.^45^ Their activity is tightly regulated by a balance of activating and inhibitory receptor signals. One such inhibitory receptor, TIGIT, is expressed on NK cells, T cells and tumour‐infiltrating lymphocytes and is known to suppress cytotoxicity and induce T‐cell dysfunction.^165^ *F. nucleatum* exploits this checkpoint through its Fap2 protein, which directly binds TIGIT and reduces NK‐cell cytotoxicity as well as T‐cell‐mediated antitumour responses.^153^ In addition, an inverse correlation between *F. nucleatum* abundance and CD3+ T‐cell density has been reported in CRC tissues, further supporting its role in suppressing adaptive immune surveillance.^151^ Another inhibitory pathway involves CEACAM1, which is expressed on NK cells and T cells and has been linked to T‐cell exhaustion and reduced IFN‐γ production.^151^, ^154^, ^166^ *F. nucleatum* has been reported to inhibit T‐cell and NK‐cell function through direct engagement of CEACAM1, providing a second mechanism by which the bacterium dampens cytotoxic immunity.^154^ Together, these observations indicate that *F. nucleatum* suppresses lymphocyte‐mediated antitumour defence through receptor‐level interference with key immune effector populations.

#### 4.3.2 Recruitment of myeloid‐derived suppressor cells

One important mechanism is the recruitment and activation of myeloid‐derived suppressor cells (MDSCs). *F. nucleatum* can promote MDSC accumulation through chemotactic signalling, and these cells in turn suppress T‐cell proliferation by depleting amino acids, up‐regulating arginase‐1 and restricting cysteine availability.^141^, ^142^, ^143^ In parallel, *F. nucleatum* has been associated with enhanced PD‐L1 and CD47 expression, potentially via MYC‐related transcriptional programs, thereby reinforcing tumour‐cell immune escape and tolerance.^44^, ^144^

Macrophage remodelling also represents a major component of *F. nucleatum*‐mediated immune suppression. Available studies indicate that *F. nucleatum* can alter macrophage infiltration patterns and favour immunosuppressive polarisation in multiple tumour contexts, thereby reinforcing cytokine production, stromal remodelling and tumour‐supportive signalling.^162^, ^167^, ^168^ This myeloid bias is especially relevant because it links bacterial persistence to durable changes in the inflammatory and immune architecture of the TME.

In addition, increased MMP‐9 expression in *F. nucleatum*‐associated tumours may contribute not only to invasion, metastasis, extracellular matrix remodelling and angiogenesis, but also to the establishment of an immune‐suppressive microenvironment.^40^, ^145^, ^146^, ^169^ Collectively, these findings indicate that *F. nucleatum* promotes cancer progression not only through direct oncogenic signalling, but also through broad immune–stromal remodelling that weakens host antitumour defence.

### 4.4 EMT, invasion, metastasis and OMV‐mediated signalling

Beyond inflammatory amplification and immune suppression, *F. nucleatum* also promotes malignant dissemination by enhancing EMT, invasion and metastatic competence across multiple tumour types. In oral squamous cell carcinoma, co‐culture with *F. nucleatum* increases invasive behaviour and up‐regulates EMT‐related genes. Mechanistically, this effect has been linked to activation of the lncRNA MIR4435‐2HG/miR‐296‐5p/Akt2/SNAI1 axis, which accelerates EMT progression.^170^, ^171^ In CRC, *F. nucleatum* likewise enhances migration through several mechanisms, including induction of IL‐8 and CXCL1, activation of autophagy via CARD3 and modulation of the miR‐1322/CCL20 and KRT7–AS/KRT7 pathways.^67^, ^88^, ^162^, ^172^ In addition, *F. nucleatum*‐infected CRC cells can release exosomes enriched in metastasis‐associated miRNAs and cytokines, resulting in increased β‐catenin, MYC, cyclin D1 and mesenchymal‐marker expression in recipient cells.^173^

OMVs appear to extend these effects beyond direct bacterial contact. As concentrated carriers of bacterial virulence factors, OMVs can deliver pro‐oncogenic cargo to host cells, alter mitochondrial dynamics, amplify inflammatory signalling and reshape the TME.^174^, ^175^, ^176^, ^177^, ^178^, ^179^ Compared with parental bacteria, OMVs may exert amplified pathogenic effects because of cargo enrichment and enhanced tissue penetration. These findings support the concept that *F. nucleatum* promotes metastasis not only through direct cell‐associated interactions, but also through vesicle‐mediated long‐range signalling.

Angiogenic remodelling further reinforces this prometastatic state. *F. nucleatum* has been shown to stimulate endothelial cells to secrete increased levels of VEGF and VEGFRs, thereby promoting endothelial activation, tumour‐cell proliferation and metastatic dissemination.^180^ In parallel, MDSCs recruited in response to *F. nucleatum* reduce T‐cell infiltration and enhance secretion of MMP‐9 and MMP‐13, which further increases angiogenesis and stromal remodelling.^181^ Taken together, these findings indicate that *F. nucleatum* facilitates metastasis through a multilayered process involving EMT induction, cytokine and exosome signalling, OMV‐mediated host reprogramming and vascular remodelling.

## 5 *F. NUCLEATUM* IN METASTATIC DISSEMINATION AND COLONISATION

The detection of *F. nucleatum* in metastatic lesions provides important evidence that its role in cancer extends beyond primary tumour growth. In CRC, *F. nucleatum* has been identified not only in primary tumours but also in liver and lymph node metastases, supporting the notion that this bacterium can persist throughout the metastatic cascade rather than acting solely at the stage of local tumour initiation.^182^ This observation is biologically significant because it suggests that *F. nucleatum* may contribute not only to the acquisition of invasive traits, but also to the establishment and maintenance of metastatic niches in distant organs.

Building on the prometastatic programs described above, *F. nucleatum* appears to facilitate metastatic dissemination through coordinated interactions with tumour cells, stromal cells and the surrounding microenvironment. In CRC, higher *F. nucleatum* levels correlate with EMT marker expression and with features of colitis‐associated cancer progression, supporting a link between bacterial abundance and invasive tumour phenotypes.^183^ Mechanistically, *F. nucleatum* has been reported to activate a TLR4‐dependent EGFR/AKT/ERK signalling axis in which TLR4/MyD88 signalling induces amphiregulin expression in cancer‐associated fibroblasts, while FadA‐mediated disruption of E‐cadherin releases β‐catenin to promote epiregulin transcription, thereby amplifying EGFR signalling in tumour cells.^181^ Rather than functioning as an isolated EMT pathway, this circuit may be viewed more broadly as a stromal–epithelial signalling loop that supports invasive growth and metastatic competence.

Metastatic promotion by *F. nucleatum* may also involve metabolic reprogramming. Cytochrome P450 enzymes, particularly CYP2J2, have been implicated in tumour progression and treatment resistance.^184^, ^185^ In CRC, *F. nucleatum* infection has been associated with elevated CYP2J2 expression and increased production of the metabolite 12,13‐EpOME, both of which are linked to enhanced invasion, migration and EMT‐like phenotypes. This effect is mediated in part through the TLR4/AKT/Keap1/NRF2 axis, which not only up‐regulates CYP2J2 but may also synergise with EGFR‐related signalling. In this framework, bacterial signalling is coupled to a prometastatic metabolic program that may further reinforce tumour plasticity in hypoxic microenvironments.^58^

Another important implication of metastatic *F. nucleatum* persistence is that the bacterium may continue to shape the biology of disseminated cancer cells after arrival at distant sites. By sustaining inflammatory signalling, stromal activation and immune dysregulation, *F. nucleatum* may help create a permissive microenvironment for metastatic outgrowth. This concept is supported by the observation that *F. nucleatum*‐associated tumours frequently display enhanced cytokine signalling, extracellular matrix remodelling and immune‐evasive features, all of which are relevant to metastatic colonisation and survival. Thus, the contribution of *F. nucleatum* to metastasis should not be viewed solely as an early invasion‐promoting event, but as a dynamic process that may also support colonisation efficiency and persistence at secondary sites. From a translational perspective, the presence of *F. nucleatum* in metastatic lesions strengthens its relevance as a biomarker‐associated organism and a potential therapeutic target. Persistent bacterial colonisation in metastatic tissue raises the possibility that *F. nucleatum* burden could help identify tumours with higher metastatic potential, poorer prognosis or altered treatment responsiveness. More broadly, these findings suggest that anti‐*F. nucleatum* strategies may need to address not only primary tumour colonisation but also bacterial persistence within disseminated or established metastatic disease.

## 6 THERAPEUTIC IMPLICATIONS

The translational relevance of *F. nucleatum* in cancer lies in its dual role as both a biomarker‐associated organism and a modifiable component of the TME. Accumulating evidence indicates that *F. nucleatum* burden is linked to prognosis, metastatic behaviour and treatment response across several malignancies, particularly CRC, oesophageal squamous cell carcinoma (ESCC) and head and neck/oral cancers. These observations suggest that *F. nucleatum* is not merely a mechanistic contributor to tumour biology, but also a clinically meaningful variable that may inform risk stratification and therapeutic design.^38^, ^41^, ^168^, ^186^

### 6.1 Biomarker potential and treatment stratification

One of the most immediate clinical implications of *F. nucleatum* research is its potential utility as a biomarker. In CRC, higher levels of *F. nucleatum* DNA in tumour tissue have been associated with shorter survival and proposed as a prognostic biomarker. In ESCC, intratumoural *F. nucleatum* levels have been linked to poor recurrence‐free survival after neoadjuvant chemotherapy, supporting its possible role in treatment stratification. In locally advanced rectal cancer, persistence of *F. nucleatum* after neoadjuvant chemoradiotherapy has been associated with higher relapse risk, suggesting that dynamic monitoring of bacterial burden may provide clinically useful information beyond baseline detection alone.^38^, ^41^, ^187^

From a practical perspective, these findings support a framework in which *F. nucleatum* assessment could be incorporated into molecular and microenvironmental profiling of tumours. Tissue‐based detection, longitudinal post‐treatment monitoring and correlation with recurrence or response may help identify patients with biologically aggressive, microbiota‐associated disease. Although standardisation of sampling methods and detection thresholds remains necessary, current data justify considering *F. nucleatum* burden as a candidate biomarker for prognosis and therapeutic decision support.^187^

### 6.2 Chemotherapy and radiotherapy resistance

The contribution of *F. nucleatum* to chemotherapy resistance has been most clearly described in CRC. Experimental studies have shown that *F. nucleatum* promotes resistance to 5‐fluorouracil and oxaliplatin by activating TLR4/MYD88‐dependent autophagy and suppressing apoptosis. Similar associations have been reported in ESCC, where high intratumoural *F. nucleatum* levels predict poorer outcomes after neoadjuvant chemotherapy, and in oral/head and neck cancer models in which *F. nucleatum*‐associated signalling reinforces survival‐promoting programs. Together, these data suggest that *F. nucleatum* may function as a microbial determinant of reduced chemosensitivity rather than simply a bystander correlated with aggressive disease.^38^, ^47^, ^168^

Radiotherapy‐related findings point in a similar direction. In rectal adenocarcinoma, patients in whom *F. nucleatum* became undetectable after chemoradiotherapy experienced significantly longer relapse‐free survival than those with persistent bacterial colonisation. Preclinical CRC studies have likewise linked *F. nucleatum* persistence with reduced radiation efficacy, sustained tumour growth and enhanced metastatic progression. These observations raise the possibility that *F. nucleatum* could serve not only as a marker of radioresistance, but also as a modifiable target whose elimination may improve locoregional control.^61^, ^187^

### 6.3 Immunotherapy response and context dependency

The relationship between *F. nucleatum* and immunotherapy is more complex and appears to be context dependent. In CRC, several studies suggest that *F. nucleatum* may enhance responsiveness to PD‐1/PD‐L1 blockade, potentially by increasing inflammatory immune‐cell infiltration and altering tumour‐cell states in ways that sensitise tumours to checkpoint inhibition. A 2021 study reported that high *F. nucleatum* levels correlated with improved response to PD‐1 blockade in CRC, and a 2024 single‐cell analysis similarly suggested that *F. nucleatum* infection may increase the abundance of T cells, NK cells and pro‐inflammatory macrophage subsets in tumours treated with PD‐L1 blockade.^188^, ^189^

In contrast, not all tumour settings show this pattern. In lung cancer, airway‐enriched Fusobacterium prior to anti‐PD‐1 therapy has been associated with poor clinical response. More recently, in head and neck squamous cell carcinoma, *F. nucleatum*‐derived OMVs were shown to promote immunotherapy resistance by inducing immunosuppressive tumour‐associated macrophage phenotypes through tryptophan‐metabolism signalling, and both *F. nucleatum* and TDO2‐related signalling showed predictive value for anti‐PD‐1 outcomes. These divergent findings indicate that the effect of *F. nucleatum* on immunotherapy cannot be generalised across cancers. Instead, its impact likely depends on tumour type, local immune architecture, strain‐level bacterial traits, polymicrobial interactions and the particular arm of immunity engaged by treatment.^168^, ^186^

This context dependency has important translational implications. Rather than treating *F. nucleatum* uniformly as “good” or “bad” for immunotherapy, future studies should determine in which tumour ecosystems it promotes productive immune activation and in which it reinforces macrophage‐ or myeloid‐dominant suppression. This distinction is especially important for microbiome‐guided precision oncology, where the same microbial signal may imply very different therapeutic strategies depending on disease context. More broadly, mechanistic studies of intratumoural microbiota have shown that local bacterial accumulation can modulate innate immune pathways such as STING and thereby alter response to immunotherapy, underscoring the need to interpret *F. nucleatum* within the larger tumour–microbiota–immunity network rather than in isolation.^79^, ^168^, ^186^

Innate immune sensing pathways may provide one mechanistic explanation for these context‐dependent immunotherapy effects. Although direct evidence that *F. nucleatum* itself activates the cGAS–STING pathway in CRC remains limited, broader intratumoural microbiota studies have shown that locally accumulated gut bacteria can modulate antitumour immunity through STING‐dependent mechanisms. For example, intratumoural accumulation of gut microbiota was reported to facilitate CD47 blockade‐mediated immunotherapy by activating STING signalling and enhancing local innate immune activation. This finding suggests that tumour‐resident bacteria may influence immunotherapy not only through systemic gut immune modulation, but also through direct remodelling of the local tumour immune microenvironment.^79^, ^190^ In this context, future studies should determine whether *F. nucleatum* or *F. nucleatum*‐associated polymicrobial communities can engage cGAS–STING‐related innate sensing in CRC and whether this pathway contributes to the divergent immunotherapy outcomes observed across tumour types.

### 6.4 Emerging therapeutic strategies targeting intratumoural *F. nucleatum*

Beyond its value as a biomarker, *F. nucleatum* is increasingly being explored as a therapeutic target. Recent preclinical work provides proof of concept that selectively reducing intratumoural *F. nucleatum* can enhance anticancer efficacy. In CRC, tumour‐targeting acidity‐responsive nanoassemblies have been developed to eliminate intratumoural *F. nucleatum* and improve treatment response, while an antibacterial sonodynamic nanoplatform was shown to suppress intratumoural *F. nucleatum*, enhance tumour apoptosis, improve sonodynamic therapy and reduce lung metastasis in orthotopic CRC models. These studies support the idea that microbiota‐directed intervention can be integrated with established antitumour modalities rather than viewed as a separate therapeutic concept.^61^, ^68^

Therapeutic vaccination strategies are also emerging. A *F. nucleatum* dendritic cell‐based vaccine has shown preclinical efficacy in CRC, and subsequent work reported that Tubeimoside I could improve the efficacy of this vaccine platform. In addition, a 2024 biomimetic antibacterial nanovaccine was reported to selectively eliminate *F. nucleatum* without disrupting intratumoural and gut microbiota broadly, while simultaneously enhancing chemotherapy efficacy and reducing metastasis in *F. nucleatum*‐infected CRC models. Taken together, these approaches suggest that future therapeutic paradigms may combine conventional cancer therapy with selective microbial eradication, microbial immunomodulation or microbiota‐aware nanomedicine.^69^, ^191^, ^192^

Although these strategies remain largely preclinical, they collectively shift the field from descriptive microbiome–cancer associations towards actionable intervention concepts. At present, the most realistic translational path is likely to involve three linked applications: using *F. nucleatum* burden to stratify risk, monitoring persistence during therapy and selectively targeting intratumoural bacteria to overcome resistance or suppress metastatic progression. The next challenge is to determine which patients, tumour types and treatment settings are most suitable for this microbiome‐guided approach.^41^, ^61^, ^68^, ^187^

## 7 GAPS AND FUTURE DIRECTIONS

Despite the rapid expansion of research on *Fusobacterium nucleatum* in cancer, several conceptual and translational gaps remain unresolved. Current evidence strongly supports a role for *F. nucleatum* in tumour colonisation, inflammatory signalling, immune modulation, treatment resistance and metastatic progression; however, many mechanistic conclusions have been derived from CRC‐focused models, and their generalisability across tumour types remains uncertain. Future work should therefore move beyond descriptive association studies and focus on resolving biological heterogeneity, establishing rigorous biomarker standards and developing selective intervention strategies that can be translated into clinical practice.^59^, ^60^

### 7.1 Resolving biological heterogeneity

A major challenge for the field is biological heterogeneity at multiple levels. First, *F. nucleatum* is not a uniform entity: subspecies‐ and clade‐level differences, including the distinction between Fna C1 and Fna C2, appear to influence niche preference, virulence and cancer relevance. Second, the effects of *F. nucleatum* vary substantially across tumour types. For example, oral‐cancer studies highlight a strong role for OMV‐mediated autophagy and metastasis, whereas gastric‐cancer work emphasises tumour‐associated neutrophil recruitment and immune evasion through NF‐κB‐related signalling. These differences suggest that future studies should explicitly distinguish shared oncogenic programs from context‐specific ones, rather than assuming that findings in CRC can be directly extrapolated to all cancers.^59^, ^60^

A related unresolved issue is the apparently contradictory role of *F. nucleatum* in immunotherapy. Available evidence indicates that the same organism may enhance or impair treatment response depending on tumour ecology, immune composition, bacterial strain and the broader polymicrobial community. Future research should therefore incorporate strain‐resolved sequencing, standardised infection models and cross‐cancer comparative analysis to define when *F. nucleatum* promotes productive antitumour immunity and when it reinforces macrophage‐ or myeloid‐dominant suppression.

### 7.2 Defining spatial, intracellular and polymicrobial ecology

Another key priority is to clarify where *F. nucleatum* resides within tumours and how that localisation shapes function. Evidence from intratumoural microbiology suggests that bacteria may persist not only on mucosal or necrotic surfaces, but also within protected niches and, in some settings, intracellularly. This distinction is likely to be biologically important because intracellular or sheltered bacteria may be more resistant to immune clearance and more capable of sustaining long‐range host–microbe signalling despite low biomass. Future studies should therefore combine spatial transcriptomics, multiplex imaging, single‐cell approaches and organoid or co‐culture systems that preserve vascular, immune and stromal complexity.^59^, ^60^

Polymicrobial interactions also remain underexplored. Increasing evidence indicates that *F. nucleatum* often acts within mixed‐species communities rather than in isolation, and these communities may amplify inflammation, alter metabolite availability and reshape immune phenotypes. Consequently, future models should examine not only *F. nucleatum* monocolonisation, but also cooperative and competitive interactions with other tumour‐associated microbes. This will be essential for understanding whether specific consortia, rather than single organisms, drive clinically meaningful phenotypes.^193^, ^194^

### 7.3 Strengthening biomarker qualification and study design

Although *F. nucleatum* has shown promise as a prognostic and treatment‐response biomarker, its clinical implementation is limited by methodological inconsistency. Across studies, sample type, tissue handling, low‐biomass contamination control, quantification thresholds and analytic pipelines vary substantially. In addition, many studies remain retrospective and are underpowered for formal biomarker qualification. Future work should prioritise harmonised protocols for tissue‐based and longitudinal detection, explicit contamination controls and prospective studies that test whether *F. nucleatum* burden adds value beyond established clinicopathologic and molecular predictors.^60^, ^194^

A particularly important next step is dynamic monitoring. The persistence or clearance of *F. nucleatum* during treatment may be more clinically informative than a single baseline measurement, especially in rectal cancer and other settings where therapy‐induced shifts in bacterial burden appear linked to recurrence risk. Accordingly, future biomarker studies should move from static presence/absence frameworks towards longitudinal, treatment‐integrated designs that capture persistence, eradication and reseeding.

### 7.4 Precision eradication and microbiome‐targeted intervention

The therapeutic targeting of intratumoural *F. nucleatum* is a particularly promising but still immature area. Bacteriophage‐based approaches are attractive because of their host specificity and potential to spare beneficial microbes. The lytic phage FNU1 was first characterised in 2019 as a *F. nucleatum*‐targeting Siphoviridae phage capable of reducing biofilm biomass and killing bacteria within biofilms. A 2023 study then examined immune effects of crude and purified FNU1 preparations, while a 2026 study reported that FNU1 could negate *F. nucleatum*‐induced cell growth, migration and chemotherapy resistance in GI cancer cells. In parallel, a 2025 study described another *F. nucleatum* phage, ØTCUFN3, that inhibited *F. nucleatum*‐induced CRC proliferation and EMT‐marker expression and reduced growth of *F. nucleatum*‐infected xenografts. Together, these studies establish proof of concept for phage therapy, but they also highlight the need for work on host range, phage cocktails, resistance emergence, delivery into tumours and efficacy in immunocompetent in vivo models.^193^, ^195^, ^196^, ^197^

Antibiotic‐based eradication strategies should also be discussed more carefully than simply advocating broad anaerobic coverage. Existing data suggest that susceptibility patterns are variable across Fusobacterium species and settings: one 2022 clinical study found all tested *F. nucleatum* isolates susceptible to the antimicrobial agents examined, whereas more recent reviews and surveillance data indicate geographic variability and emerging resistance patterns involving agents such as benzylpenicillin, clindamycin and metronidazole across oral anaerobes and some fusobacterial isolates. Earlier work also documented β‐lactamase‐producing oral *F. nucleatum* subspecies. Therefore, the future direction is not indiscriminate antibiotic use, but rather β‐lactamase‐aware, susceptibility‐guided and microbiota‐sparing anti‐anaerobic strategies, ideally coupled with local delivery or tumour‐targeted platforms to minimise collateral ecological disruption.^198^, ^199^, ^200^, ^201^

More broadly, precision eradication may ultimately require combining several approaches: selective phages, nanoplatform‐based delivery, immune modulation and treatment‐timed microbial clearance. A central unanswered question is whether eliminating *F. nucleatum* will be most effective before therapy, during therapy to reverse resistance or after therapy to reduce metastatic persistence and relapse. Resolving that question will require longitudinal intervention studies rather than endpoint‐only tumour measurements.^196^, ^197^

### 7.5 Exposure heterogeneity and molecular pathological epidemiology

A final gap concerns patient‐level heterogeneity in exposure history. Oral health, periodontal disease, diet, antibiotic exposure, smoking, metabolic status and host immune condition may all influence *F. nucleatum* carriage, translocation, persistence and downstream tumour phenotypes. Environmental and lifestyle factors may also influence intestinal cellular pathophysiology by altering mucosal barrier integrity, epithelial turnover, local inflammatory tone, immune–epithelial crosstalk and microbial translocation, thereby shaping tumour microbial burden and downstream immune–molecular phenotypes. Yet these exposure variables are rarely integrated with tumour microbial burden, immune architecture, personalised tumour tissue biomarkers and molecular pathology in the same study. The field of molecular pathological epidemiology (MPE), including microbiology‐informed MPE frameworks, provides a useful conceptual model for linking environmental and lifestyle factors with tumour molecular features, immune‐cell states, microbiota, personalised biomarkers, therapy response/resistance and clinical outcomes.^202^, ^203^ Applying this framework to *F. nucleatum* research could help explain why patients with ostensibly similar tumours show markedly different microbial profiles, prognoses or treatment responses.

In summary, the next phase of *F. nucleatum* research should prioritise five goals: resolving strain‐ and tumour‐specific heterogeneity, defining spatial and intracellular ecology, qualifying biomarkers using rigorous prospective designs, developing precision microbial‐eradication strategies and integrating exposure data within transdisciplinary frameworks such as MPE. Addressing these issues will be essential if the field is to move from compelling association studies towards microbiome‐guided precision oncology.^196^, ^202^

## 8 CONCLUSIONS

In summary, *Fusobacterium nucleatum* has emerged as an important tumour‐associated bacterium that actively contributes to cancer progression rather than merely existing as a passive intratumoural inhabitant. Current evidence indicates that *F. nucleatum* promotes malignant phenotypes through coordinated effects on tumour colonisation, inflammatory signalling, immune evasion, metastatic dissemination and treatment response, although these effects appear to be context dependent across different cancer types. Beyond its mechanistic significance, *F. nucleatum* is increasingly relevant from a translational perspective as both a biomarker‐associated organism and a potential therapeutic target. Future studies should therefore focus on clarifying its strain‐level and tumour‐specific heterogeneity, defining its spatial and intracellular ecology and developing standardised detection and selective targeting strategies, so that insights into *F. nucleatum* biology can be more effectively translated into microbiome‐guided precision oncology.

## AUTHOR CONTRIBUTIONS

F. Z. and R. A. composed the main text, figures and tables. S. Y., Y. M., W. L., C. S., X. X. and J. Z. reviewed and refined the work. All authors have read and approved the manuscript for publication.

## CONFLICT OF INTEREST STATEMENT

The authors declare no conflicts of interest.

## ETHICS STATEMENT

The authors have nothing to report.

## CONSENT

The authors have nothing to report.

## Data Availability

No new datasets were generated or analysed in this review. All data and evidence discussed in this article were derived from previously published studies, which are cited in the relevant sections and figure legends. The schematic figures were created based on literature synthesis and do not contain newly analysed public datasets. Publicly available resources, when discussed, are cited with their original publications.
